# Dialysis Dependence Is Associated With Significantly Increased Odds of Perioperative Adverse Events After Geriatric Hip Fracture Surgery Even After Controlling for Demographic Factors and Comorbidities

**DOI:** 10.5435/JAAOSGlobal-D-19-00086

**Published:** 2019-08-06

**Authors:** Taylor D. Ottesen, Alp Yurter, Blake N. Shultz, Anoop R. Galivanche, Cheryl K. Zogg, Patawut Bovonratwet, Lee E. Rubin, Jonathan N. Grauer

**Affiliations:** From the Department of Orthopaedics and Rehabilitation, Yale University School of Medicine, New Haven, CT.

## Abstract

Previous studies evaluating the risk of perioperative adverse events after hip fracture surgery for dialysis-dependent patients are either institutional cohort studies or limited by patient numbers. The current study uses the National Surgical Quality Improvement Program database's large national patient population and 30-day follow-up window to address these weaknesses.

**Methods:**

National Surgical Quality Improvement Program databases (2006 to 2016) were queried for patients aged 60 years or older who underwent hip fracture surgery. Differences in 30-day outcomes based on preoperative dialysis dependence were compared using risk-adjusted logistic regression and coarsened exact matching for adverse events, need for revision surgery, readmission, and mortality. The proportion of adverse events that occurred before versus after discharge was also assessed.

**Results:**

A total of 288 dialysis-dependent and 16,392 non–dialysis-dependent patients met the inclusion criteria. Matched populations controlling for demographic factors (ie, age, sex, body mass index, and functional status) and overall health (American Society of Anesthesiologists class) found dialysis-dependent patients to be associated with significantly greater odds of any adverse event (odds ratio [OR] = 1.90), major adverse event (OR = 1.77), and unplanned readmission (OR = 2.48). Increased odds of minor adverse event (OR = 1.05), return to the operating room (OR = 1.66), and death (OR = 1.42) within 30 postoperative days were also found but were not statistically significant.

**Discussion:**

Even after controlling for demographics and health status, geriatric dialysis patients undergoing surgery for hip fracture are at significantly greater odds of adverse outcomes. Because of increased risks for geriatric dialysis patients undergoing surgery for hip fracture, surgical caution, patient counseling, and heightened surveillance must be observed throughout the perioperative period for this fragile population. Furthermore, hospitals and physicians must take the increased risks associated with dialysis into account when considering bundled payment reimbursement strategies and resource allocation for hip fracture care.

The incidence of patients with end-stage renal disease (ESRD) has increased significantly over the years, with 120,688 new cases of ESRD being reported in the United States in 2014 alone.^1^ Dialysis continues to be the predominant treatment modality for ESRD, and the number of dialysis-dependent patients has paralleled the rise in ESRD cases. Over the past decade, the dialysis-dependent population has increased 24%, reaching an all-time high of 113,944 incident cases in 2013.^2^ The increased prevalence of dialysis is particularly relevant to orthopaedic surgeons, given its negative associations with bone health, osteoarthritis, amyloidosis, and metabolic abnormalities that can increase the risk for fractures and decrease potential for healing after such injuries.^3,4^

Hip fracture among elderly patients is a common but dangerous occurrence, with more than 250,000 patients being admitted annually in the United States^5^ and a 1-year mortality rate between 20% and 35%.^5,6^ In addition, annual costs of caring for those with hip fractures are estimated to be as high at $15 billion per year, creating a significant burden on healthcare costs. The incidence and costs are expected to rise because of the aging population of the United States, with projections showing a quadrupling of patients older than 85 years by 2050.^7,8^

Hospitals and legislatures have recognized the need for optimizing patient outcomes as a method of cost minimization. For example, the Patient Protection and Affordable Care Act sought to decrease costs through the creation of the Hospital Readmission Reduction Program, which penalizes hospitals who do not meet pre-established markers for acceptable readmission rates.^9^ Furthermore, the Bundled Payments for Care Initiative (BPCI) created by the Center for Medicare and Medicaid Services (CMS) eschews traditional fee-for-service reimbursement in favor of increasing provider and hospital accountability for patient outcomes.^10^ To facilitate the optimization of patient outcomes, studies of complications after common procedures in high-risk, high-cost populations such as hip fracture repair in dialysis-dependent patients are necessary.

Currently, the literature regarding surgical outcomes in dialysis-dependent hip fracture patients is limited by small populations not exceeding more than 62 patients.^11,12,13,14,15,16,17,18,19,20^ Unfortunately, the limited statistical power of these studies constrains the utility and generalizability of the perioperative data. Furthermore, although these studies suggest that dialysis patients fare worse after hip fracture, there is little mention of whether this is due to dialysis as an independent risk factor or whether the negative outcomes are driven by overall health deficits in this population. In contrast, one larger study using the National Trauma Database contained larger numbers but the database is limited to the inpatient stay, and the data sets are not necessarily nationally representative, especially as higher-level trauma centers are excluded from the database outcomes.^21,22^

To address the above-noted limitations, the current study used the American College of Surgeons National Surgical Quality Improvement Program (NSQIP) database. This database is a large, national database that provides 30-day outcomes and has been shown to be robust in performing risk-adjusted outcomes analysis.^23,24^ With these factors in mind, the aim of the current study is to compare perioperative outcomes and complications after hip fracture in dialysis-dependent and non–dialysis-dependent cohorts in the NSQIP database through multivariable regression analysis of coarsened exact matching (CEM) samples.

## Methods

### Data Source and Study Cohort

NSQIP contains information on >150 variables abstracted directly from patient charts by trained clinical reviewers at participating hospitals across the United States.^25^ As of 2016, it contained data from more than 600 member hospitals and included variables addressing patient demographics and comorbidities, perioperative factors, and outcomes (ie, complications, readmission, and mortality) reported within 30 postoperative days, regardless of discharge status. Interrater reliability for information abstraction from patient records is reported to be greater than 98%.^25^

The current study queried 2005 to 2016 NSQIP data for all patients aged 60 years and older undergoing isolated hip fracture repair (CPT codes: 27236, 27244, and 27245). Patients were excluded if they presented with an *International Classification of Diseases, 9th or 10th revision*, diagnosis code for infection, tumor, or emergency. Covariate information obtained from NSQIP for use in this study included age, sex, body mass index (BMI) (kg/m^2^), functional status before injury, and American Society of Anesthesiologists' (ASA) classification score. Receipt of dialysis, defined by NSQIP as any patient currently requiring or receiving dialysis before surgery, was the primary explanatory variable.

### Perioperative Adverse Events and Secondary Outcome Measures

The primary outcome variable for the study was defined as the occurrence of any perioperative any adverse event (AAE). The occurrence of adverse events was abstracted from NSQIP based on variables reported within 30-day of index operation.

Any adverse event (AAE) was noted if there was a reported occurrence of either a severe adverse event (SAE) or minor adverse event (MAE). SAE included documented occurrence of deep surgical site infection, sepsis, failure to wean from a ventilator within 48 hours, need for reintubation, renal failure, thromboembolic event (deep vein thrombosis/pulmonary embolism), cardiac arrest, myocardial infarction, or cerebrovascular accident (stroke). MAE included superficial surgical site infection, wound dehiscence, pneumonia, urinary tract infection, or postoperative renal insufficiency.

Additional secondary outcome measures were also assessed, including (1) the need for the patient to return to the operating room, (2) readmission (reported in NSQIP from 2011 onward), and (3) mortality (evaluated independent of other adverse events) within 30 days of index operation.

### Statistical Analyses

Primary and secondary outcome differences between patients who received and who did not receive dialysis were compared using chi-squared tests and unadjusted (univariate) logistic regression. Risk-adjusted differences were further compared using multivariable logistic regression, accounting for the influence of age, sex, BMI, functional status, and ASA classification.

To account for the relatively small sample size among dialysis patients and ensure the consistency of the reported multivariable model effects, risk-adjusted differences were also compared using logistic regression within CEM cohorts for each outcome. CEM, like other forms of matching, is designed to reduce underlying confounding differences between explanatory variable groups (ie, between patients who did and did not receive dialysis) by identifying patients who are otherwise similar with respect to the indicated covariates. CEM specifically functions by temporarily coarsening continuous covariates (eg, age) into predetermined set width groups and matching patients based on the coarsened bins, in addition to directly matching based on categoric variables (eg, sex). Thus, compared with other matching techniques, CEM is more invariant to measurement error, ensuring that reducing balance in one covariate has no effect on other included covariates, balancing nonlinearities, and being extremely computationally efficient.^26–28^ It does not require iterations for balance checking and rematching or use of a separate procedure for estimation. Moreover, because CEM is from the family of monotonic imbalance bounding methods, matching is precise on coarsened and included categoric variables, making further adjustment on the same variables redundant (ie, risk adjustment on matched covariates is not needed).

Akin to the risk-adjusted logistic regression models, patients in CEM cohorts were matched on differences in age, sex, BMI, functional status, and ASA classification. All patients in each explanatory group for each outcome meeting matching parameters were retained (1:1 matching for nondialysis versus dialysis receipt). To assess for the success of the CEM procedure, overall differences between matched and nonmatched cohort sets for each outcome were compared using the L1 statistics technique.^26–28^ The L1 statistic is a comprehensive measure of global imbalance between groups. Values close to one indicate complete separation (ie, the groups are not matched), while values close to zero indicate perfect balance (ie, no difference between groups). Successful CEM should result in a reduction of the before matching L1 value. A direct comparison of covariate distributions after matching between dialysis and nondialysis patients for the AAE cohort was also included and is presented in Table 1. Similar methodology has been previously used in surgical studies in other fields.^29,30^

**Table 1:**
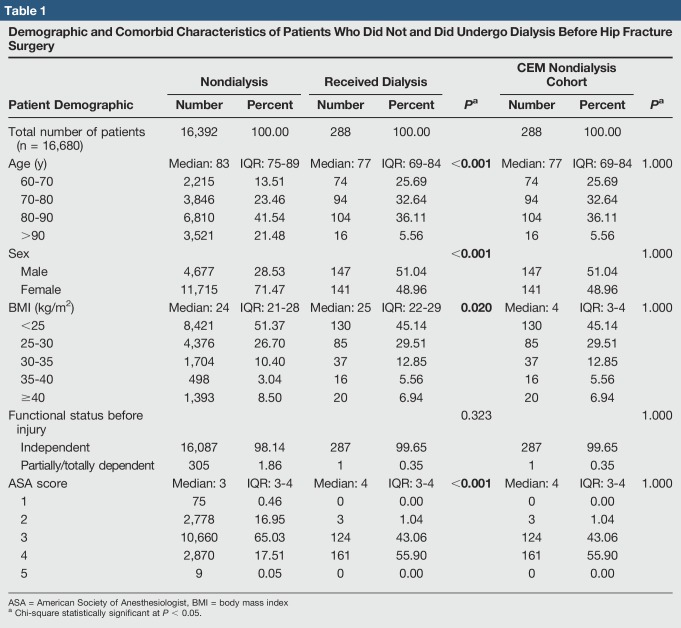
Demographic and Comorbid Characteristics of Patients Who Did Not and Did Undergo Dialysis Before Hip Fracture Surgery

Subanalyses compared differences in AAE based on when reported events occurred—before versus after discharge—between patients who received and did not receive dialysis. The timing of adverse events was determined in acute coronary syndrome NSQIP by comparing the number of postoperative days that an adverse event was recorded to the corresponding patient's reported index hospital length of stay.

All analyses and data management were conducted using Stata Statistical Software: Release 14.2 (StataCorp LP, College Station, TX). Two-sided *P* values <0.05 were considered statistically significant. Our institution's Human Investigation Committee (Institutional Review Board) determined the study exempt.

## Results

### Patient Population

A total of 288 dialysis and 16,392 nondialysis patients who underwent hip fracture surgery met the study inclusion criteria. The nondialysis group was older in age compared with the dialysis group (median age: 83 versus 77 years, *P* < 0.001). The nondialysis group also had a lower percentage of male patients (28.53% versus 51.04%, *P* < 0.001), lower ASA score (3 versus 4, *P* < 0.001), and a lower BMI (24 versus 25 kg/m^2^, *P* = 0.020) (Table 1). All results were considered statistically significant for *P* values less than 0.05 and subsequently bolded in all tables.

To control for differences in preoperative characteristics and group size, a CEM group of 288 nondialysis patients was chosen from the total population to generate a comparable cohort. After CEM, age, sex, BMI, functional status before injury, and ASA class were statistically similar in both cohorts (prematching AAE L1 = 0.547; postmatching AAE L1 = 0.247) (Table 1).

### Surgical Outcomes

Rates of 30-day adverse outcomes of dialysis-dependent and nondialysis patients are presented in Table 2. On univariate analysis, dialysis-dependent patients were found to have a greater odds of AAE (odds ratio [OR] = 2.17, 95% confidence interval [CI] 1.71 to 2.75, *P* < 0.001), SAE (OR = 2.36, 95% CI 1.83 to 3.05, *P* < 0.001), return to the operating room within 30 days (OR = 2.04, 95% CI 1.16 to 3.60, *P* = 0.013), readmission to the hospital within 30 days of the operation (OR = 2.45, 95% CI 1.79 to 3.33, *P* < 0.001), and mortality within 30 days of the operation (OR = 2.29, 95% CI 1.59 to 3.32, *P* < 0.001) (Table 2).

**Table 2:**
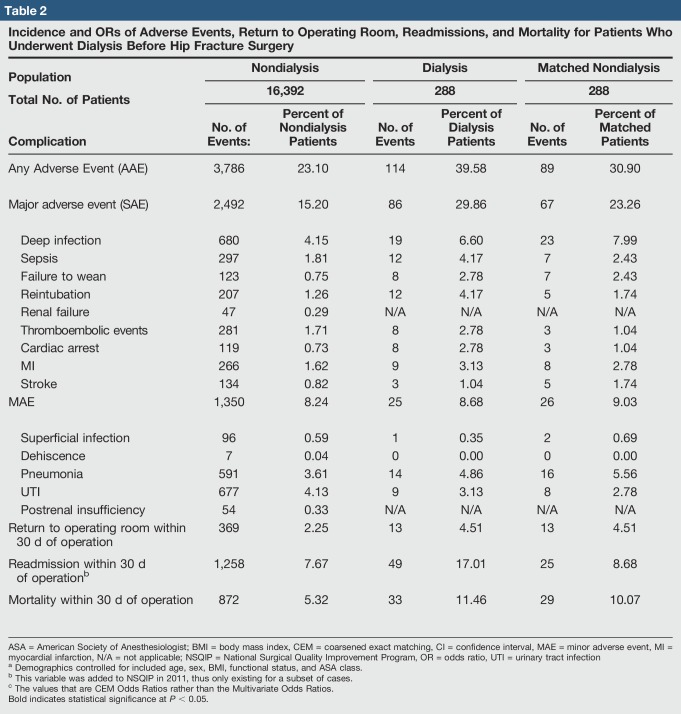

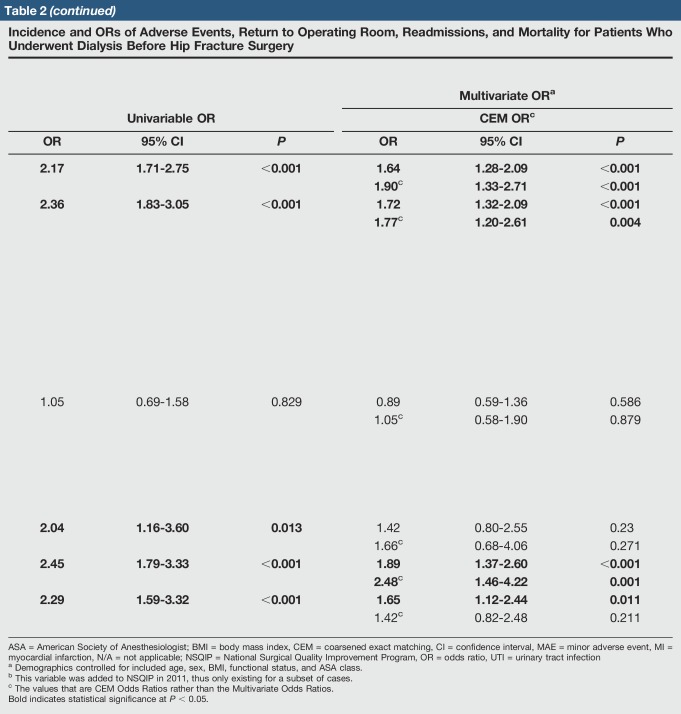
Incidence and ORs of Adverse Events, Return to Operating Room, Readmissions, and Mortality for Patients Who Underwent Dialysis Before Hip Fracture Surgery

Because of preoperative differences in cohort demographics, a multivariable logistic regression controlling for age, sex, BMI, preoperative functional status, and ASA class was performed. After controlling for these preoperative demographic and overall health factors, dialysis-dependent patients remained more likely to experience AAE (OR = 1.64 times, 95% CI 1.28 to 2.09, *P* < 0.001), SAE (OR = 1.72, 95% CI 132 to 2.09, *P* < 0.001), hospital readmission (OR = 1.89, 95% CI 1.37 to 2.60, *P* < 0.001), and mortality within 30 days of the operation (OR = 1.65, 95% CI 1.12 to 2.44, *P* = 0.011) (Table 2).

**Figure 1:**
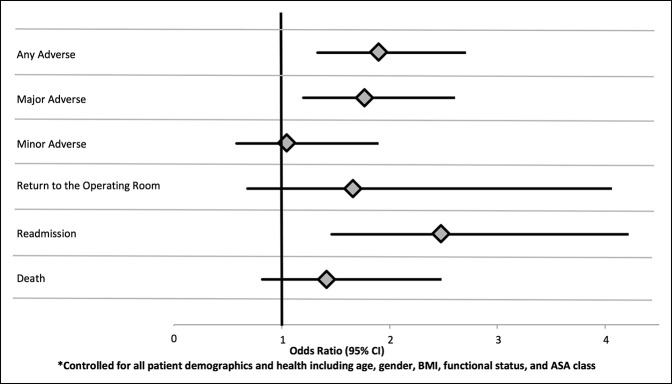
Multivariate odds ratio of dialysis patients relative to coarsened exact matching nondialysis cohorts for adverse events and binomial outcomes*.

Finally, a multivariable comparison of dialysis patients and a selection of CEM nondialysis patients was performed. In this analysis, patients on dialysis were more likely to experience AAE (OR = 1.90, 95% CI 1.33 to 2.71, *P* < 0.001), SAE (OR = 1.77, 95% CI 1.20 to 2.61, *P* = 0.004), return to the OR (OR = 1.66, 95% CI 0.68 to 4.06, *P* = 0.271), and experience readmission within 30 days of the operation (OR = 2.48, 95% CI 1.46 to 4.22, *P* = 0.001), (Table 2 and Figure 1).

### Pre- versus Post-discharge Outcomes

**Figure 2:**
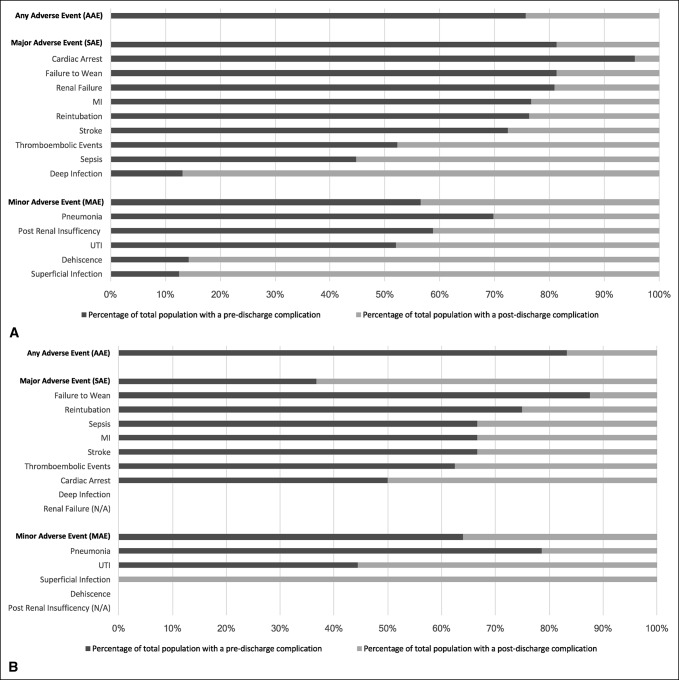
**A**, Comparison of the percentage of complications predischarge versus postdischarge for nondialysis patients. **B**, Comparison of the percentage of complications predischarge vs. postdischarge for dialysis patients.

The timeline of postoperative complications in both patient cohorts was further analyzed. A sizable percentage of AAEs, SAEs, and MAEs occurred postdischarge. In nondialysis patients, 24.38% of AAEs occurred postdischarge (Table 3 and Figure 2, A), whereas 16.67% occurred postdischarge in dialysis patients (Table 3 and Figure 2, B). In general, SAEs were more likely than MAEs to occur before discharge, although several types of SAEs and MAEs had considerable postdischarge incident rate.

**Table 3:**
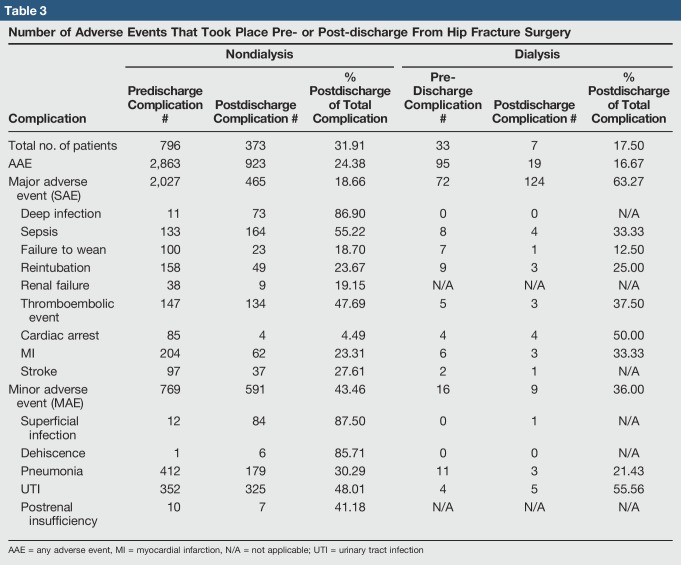
Number of Adverse Events That Took Place Pre- or Post-discharge From Hip Fracture Surgery

## Discussion

The dialysis-dependent population is continuing to rise in the United States. This is particularly relevant to orthopaedic surgeons because of an increased risk for fractures and decreased potential for healing after such injuries in this population.^3,4^ Although studies analyzing postoperative complications in hip fracture patients receiving dialysis have been conducted, they have typically focused on long-term outcomes and have been limited by sample size and/or an absent control group.^13,14,15,16,17,18,19,21^ To address these shortcomings, the current study assessed the association of dialysis dependence with perioperative adverse events after hip fracture surgery using the NSQIP database.

The current study identified 289 geriatric dialysis-dependent patients who had undergone hip fracture surgery. This cohort was significantly larger than those of previous studies examining dialysis dependence as an independent prognosticator of postoperative outcomes, in which populations have ranged from 8 to 62 patients.^11,12,13,14,15,16,17,18,19,20^ With the increased power of the current study, univariate, multivariate, and matched multivariate analyses were used to compare geriatric hip fracture patients with and without preoperative dialysis.

The univariate analyses demonstrated that dialysis dependence was significantly associated with an increased risk of adverse events. These findings are generally consistent with the notion that dialysis patients exhibit multiorgan dysfunction,^13,31^ increasing the risk for postoperative complications.^32^ Specifically, anemia, electrolyte/fluid imbalance, and cardiovascular events deriving from chronic renal failure escalate the risk of surgical mortality and morbidity. In addition, dialysis patients typically exhibit malnutrition, coagulopathy, and compromised immunity, increasing susceptibility to wound complications and prosthetic infection.^33^ Indeed, the current dialysis cohort had a significantly higher percentage of patients having an ASA score of three or greater, confirming greater disease burden.

To determine the role of dialysis dependence in perioperative outcomes, multivariate analysis controlling for demographics and overall patient health condition was conducted. The dialysis-dependent cohort was found to be at an increased risk for AAE, major adverse event, unplanned readmission, and mortality within 30 postoperative days. To strengthen the analysis and account for the disparate sizes of the dialysis-dependent and nondialysis cohorts, CEM was performed. The dialysis sample similarly showed an increased risk of all the aforementioned outcomes relative to the non–dialysis-matched sample with the exception of mortality.

These findings underscore the significance of dialysis as an independent predictor of postoperative complications and readmission, with dialysis-dependent patients being at increased odds of adverse events even when compared against a demographically and health status–matched nondialysis cohort. These results are particularly relevant to the evolving reimbursement system, as the CMS, established under the Patient Protection and Affordable Care Act of 2010, has been experimenting with various bundled payment schemes to minimize costs of orthopaedic procedures.^34^

In 2013, The Bundled Payments for Care Improvement (BPCI) Initiative was passed. This was a voluntary 3-year program, which demonstrated cost savings and subsequently led to the passage of the Comprehensive Care for Joint Replacement program (CJR) in 2016.^35^ The CJR is mandatory for all lower extremity joint arthroplasties in 67 geographical locations.^36^ Notably, although the CJR risk adjusts for nonelective fractures, research suggests that compensations for complex patient populations are likely inadequate.^36–38^ These higher-risk populations, as seen in the present analysis for dialysis patients, are far more likely to have an adverse event or readmission and therefore use more resources and costs for their treatment. As this risk factor is not modifiable through any known means, the result is that the patient's baseline health at the time of their presentation with an acute hip fracture has a tremendously negative impact, regardless of how well the surgery was performed.

More recently, the CMS approved the Surgical Hip and Femur Fracture Treatment Program (SHFFT), which began July 2017.^36^ However, the American Academy of Orthopaedic Surgeons has already expressed concern over inadequate risk adjustment and outcome measures.^36^ The current study brings meaningful data to the discussion of risk-adjusted reimbursement for management of higher-risk patients, as dialysis dependence alone was found to be associated with over a two-fold risk of 30-day readmission and a nearly two-fold risk for AAE or a major adverse event.

The current study does have limitations. Mostly, these are inherent to the limitations of the data set itself. NSQIP does not provide long-term follow-up or surgery-specific outcomes. In addition, NSQIP does not specify cumulative dialysis duration or type of dialysis (hemodialysis versus peritoneal dialysis), which could be pertinent to patient survival and complication rates.^39^

To our knowledge, this is the most comprehensive study analyzing the relationship between dialysis dependence and extensive perioperative adverse events in dialysis-dependent hip fracture patients. The strength of the current study derives from the quality of the NSQIP database, which contains prospectively collected, robust, validated data that is nationally representative and follows patients for 30 days.^40^

In summary, dialysis dependence is independently associated with perioperative adverse events in patients undergoing hip fracture surgery. These findings highlight the importance of preoperative patient counseling, surgical caution, and heightened surveillance throughout the perioperative period in this high-risk population. Furthermore, hospitals, physicians, and lawmakers should take these data into account when developing bundle payment reimbursement strategies.
